# Standardized surveillance of blood culture contamination rates: moving toward a universal standard

**DOI:** 10.1128/jcm.00981-25

**Published:** 2025-09-05

**Authors:** Fizza Manzoor, Sarah E. Turbett

**Affiliations:** 1 Division of Infectious Diseases, Department of Medicine, Massachusetts General Hospital 2348, Boston, Massachusetts, USA; 2 Harvard Medical School 1811 , Boston, Massachusetts, USA; 3 Department of Pathology, Massachusetts General Hospital 2348, Boston, Massachusetts, USA; Endeavor Health, Evanston, Illinois, USA

## Abstract

Blood culture contamination (BCC) is a major healthcare challenge with no standardized process to monitor and report rates by individual healthcare facilities in the United States. A new study by V. Fabre, Y.- J. Hsu, K. C. Caroll, A. M. Milstone, et al. (J Clin Microbiol 63:e0053025, 2025, https://doi.org/10.1128/jcm.00530-25) characterizes blood culture contamination rates, surveillance methods, reporting practices, and preventative strategies across 52 centers in the United States. The study highlights a need for standardized surveillance metrics and target thresholds within and across hospitals to help facilitate accurate benchmarking, provide feedback on existing processes, support antimicrobial and diagnostic stewardship efforts, and allocate resources to reduce BCC events.

## COMMENTARY

Blood culture contamination (BCC) remains an ongoing healthcare quality and patient safety challenge despite sustained efforts to reduce contamination rates. A single false positive blood culture contaminated with skin flora is associated with longer hospital stay, delayed or missed diagnoses, increased use of unnecessary antibiotics and their related complications, and an estimated $2,923–$5,812 in additional hospital costs (1, 2). Additionally, BCC increases recollection rates, which can waste limited resources during times of limited supply, such as the recent 2024 Becton Dickinson blood culture bottle shortage in the United States (3, 4). Contamination is influenced by several factors including operator technique, phlebotomy site (intravascular catheter vs venipuncture), antisepsis choice, initial specimen diversion, patient co-morbidities, collection environment and patient census, and clinician staffing (5). As a result, surveillance and prevention of BCC requires a robust multidisciplinary and system-wide effort to develop, implement, and audit effective policies, programs, and interventions (6).

Clinical laboratories in the United States are required to maintain blood culture quality control procedures by accreditation agencies, but there is no universal process to monitor and report BCC rates (7). Currently, different BCC criteria are proposed by the College of American Pathologists (CAP), the Clinical and Laboratory Standards Institute (CLSI), the American Society of Microbiology (ASM) and the Centers for Disease Control and Prevention (CDC), but use of these definitions by individual centers is highly variable (8–10). Therefore, the lack of standardized BCC surveillance, defined national targets, and subsequent accurate hospital benchmarking makes it challenging to perform data-informed quality and safety improvements, auditing of blood culture collection protocols, and implementation of education initiatives and interventions.

A new study by Fabre et al. describes a retrospective analysis of 362,078 blood cultures across 52 acute care hospitals in the United States over a 3-year period (2019–2021) to identify practices and trends in BCC rates and surveillance methods (11). The investigators calculated BCC rates using standard definitions outlined by CAP and CLSI, in addition to the expanded commensal list outlined by the National Healthcare Safety Network (NHSN). They also collected additional data on blood culture operational practices and quality indicators using a detailed questionnaire completed by a multidisciplinary team of specialists in infection prevention, antimicrobial stewardship, and microbiology at each hospital. The investigators further used multivariable regression models to adjust for factors that might be associated with BCC rates.

The investigators found variability in the way hospitals defined BCC, with most hospitals using CAP criteria (65.4%), followed by NHSN skin commensal criteria (17.3%) and other locally defined criteria (17.3%). The overall median blood culture positivity, single blood culture, and central catheter blood culture rates were 4.54% (IQR: 0–9.09%), 4.72% (IQR: 1.61–11.11%), and 2.97% (IQR: 0–11.38%), respectively. There was significant variation in BCC thresholds ranging from ≤1% to 3%, with 10% of centers having no established BCC threshold. Most hospitals (88%) did not have thresholds for single blood cultures; nearly all hospitals (99%) processed blood culture bottles regardless of blood volume per CLSI and ASM recommendations (9, 10). Additionally, the specific blood culture quality indicators used by laboratories were also diverse: 39% of centers tracked blood culture positivity, 21% tracked single blood culture rates, and 15% tracked blood cultures drawn from central catheters. Finally, although blood quality indicators were collected by the laboratories, dissemination of this information to key stakeholders (phlebotomy, infection prevention, antimicrobial stewardship, hospital quality teams, and inpatient units) was inconsistent and often incomplete, with only 33% and 19% of laboratories reporting blood culture quality indicators by unit and patient population, respectively.

BCC rates were consistently higher in critical care units compared to wards but were similar across the same setting when different BCC definitions were used. For example, BCC rates in critical care units were similar when using CAP (1.38% mean rate, 95% CI: 1.32–1.45%) and CLSI (1.42% mean rate, 95% CI: 1.36–1.49%) criteria, with a slight increase after application of NHSN criteria. A similar trend was found among BCC rates on the wards when using CAP (0.96% mean rate, 95% CI: 0.93–1.00%) and CLSI (1.03% 95% CI: 0.98–1.07%) criteria. Higher contamination rates were associated with higher rates of catheter-associated bloodstream infections (CLABSIs) in critical care units (adjusted IRR: 1.10, 95% CI 1.03–1.18, *P* = 0.008). Conversely, lower contamination rates were associated with blood culture volume monitoring (adjusted OR: 0.26, 95% CI: 0.11–0.63, *P* = 0.003), sharing blood culture data with the units (adjusted OR: 0.19, 95% CI: 0.06–0.62, *P* = 0.006), and using an electronic decision support tool to limit central catheter blood culture draws (adjusted OR: 0.12, 95% CI: 0.04–0.31). Finally, an analysis of 22 hospitals showed no association between contamination rates and intravenous vancomycin use (adjusted IRR: 1.01, 95% CI: 1.00–1.02, *P* = 0.184 in critical care units and 1.01, 95% CI: 1.00–1.02 in wards, *P* = 0.270).

This study makes several important contributions. First, it emphasizes the pressing need for standardized blood culture quality procedures and surveillance practices within and across hospitals. Standardized surveillance measures, including universal BCC definitions and thresholds, uniform blood culture quality indicators, and consistent reporting strategies within centers are necessary to accurately monitor rates across emergency department and inpatient units, as BCC rates historically vary across these locations (12, 13). In fact, the investigators found that although hospitals were tracking BCC rates, there was significant variability in how these rates were calculated (hospital vs unit vs patient population). Furthermore, despite collecting BCC rates and other varied blood culture quality indicators, over one-third of hospitals did not share these data with inpatient units despite the association between feedback and lower contamination rates. Timely standardized feedback is needed to appropriately allocate resources (education, clinical decision support, collection and disinfection protocols, and specimen diversion) to areas of highest need to maximize their clinical benefits.

Second, standardized blood culture surveillance across hospitals would pave the way for more accurate benchmarking. The investigators found that there was no universal BCC target threshold across institutions despite CDC and CLSI best practice recommendations for a target of ≤1%. A consensus BCC threshold is not only important for standardizing quality but could also be a step towards potential pay-for-performance incentives that could help invest in infrastructure to support best practices for blood culture collection, in addition to tools to prevent and more rapidly identify BCC, like specimen diversion devices and rapid molecular diagnostic tests from positive blood cultures (6). Third, this study demonstrates that despite some variation in calculated BCC rates when using different criteria, the overall rates remained generally similar, demonstrating the feasibility of developing and implementing a universal surveillance definition with limited impact on individual hospital BCC rates.

This study also highlights that there are several barriers to implementation of blood culture quality indicators even if a standardized national surveillance metric was readily used. Accurate tracking of contamination rates requires robust digital infrastructure to support detailed data analysis and reporting that may not be available in all settings. For example, the investigators found only 15% of hospitals in this study tracked blood culture draws from central catheters despite the association between BCC and CLABSI rates. Effective implementation of both new and existing standardized protocols is also highly resource intensive (5, 14). Central line BCC rates were consistently higher in critical care units despite nearly all centers having formal guidance on collection processes, demonstrating opportunities to address gaps in prevention and implementation. Despite these barriers, working toward a universal surveillance definition is the first step toward effective prevention and performing root cause analyses for improving existing processes.

The study by Fabre et al. also has important limitations. First, it was based primarily at large academic medical centers in the United States, where blood culture collection protocols, resources, and clinical care can differ significantly from other centers, limiting the generalizability of their findings. Second, the study interval overlapped with the COVID-19 pandemic, which has been associated with higher rates of BCC, but the investigators included the pandemic years as a factor in their risk-adjusted estimates (15). Third, the investigators did not evaluate BCC rates in emergency departments which often have higher contamination rates compared to other hospital areas due to higher staff turnover, patient volumes, and time constraints (16). Finally, although this study sheds some light on the association between BCC and antimicrobial and diagnostic stewardship, conclusions are limited based on incomplete data from the survey. For example, less than half of the participating hospitals had data on intravenous vancomycin use, and only half had indications for blood culture collection. The investigators also did not comment on the availability and impact of rapid molecular panels from positive blood cultures that could allow for earlier identification of contaminants and antibiotic discontinuation. Therefore, while the authors did not find a significant association between contamination rates and antibiotic use, additional research is needed to further explore these findings.

In summary, the study by Fabre et al. is a timely addition to the literature on blood culture practices and BCC surveillance. More effective BCC surveillance and prevention measures are necessary to pave the way for leveraging contamination rates as an independent healthcare quality and safety metric and to improve the validity of existing metrics that rely on accurate blood culture information like CLABSI and hospital-onset bacteremia (17). Future steps might include automated BCC surveillance using detailed electronic laboratory and clinical data, instead of manual calculation and reporting of rates by individual units and hospitals. A standardized definition for BCC surveillance and a universal BCC threshold would significantly help advance improvements in hospital and system-wide quality processes, patient safety, and health system efficiency.
